# SemEHR: A general-purpose semantic search system to surface semantic data from clinical notes for tailored care, trial recruitment, and clinical research*

**DOI:** 10.1093/jamia/ocx160

**Published:** 2018-01-19

**Authors:** Honghan Wu, Giulia Toti, Katherine I Morley, Zina M Ibrahim, Amos Folarin, Richard Jackson, Ismail Kartoglu, Asha Agrawal, Clive Stringer, Darren Gale, Genevieve Gorrell, Angus Roberts, Matthew Broadbent, Robert Stewart, Richard JB Dobson

**Affiliations:** 1 Department of Biostatistics and Health Informatics, Institute of Psychiatry, Psychology and Neuroscience, King’s College London, London, UK; 2 School of Computer and Software, Nanjing University of Information Science and Technology, Nanjing, China; 3 National Addiction Centre, Institute of Psychiatry, Psychology and Neuroscience, King’s College London, London, UK; 4 Centre for Epidemiology and Biostatistics, Melbourne School of Population and Global Health, University of Melbourne, Australia; 5 Farr Institute of Health Informatics Research, University College London, London, UK; 6 InterDigital Europe, London, UK; 7 King’s College Hospital NHS Foundation Trust, London, UK; 8 Department of Computer Science, University of Sheffield, Sheffield, UK; 9 South London and Maudsley NHS Foundation Trust, London, UK; 10 Psychological Medicine, Institute of Psychiatry, Psychology and Neuroscience, King’s College London, London, UK

**Keywords:** secondary use of EHR, information extraction, NLP, semantic search, ontology, FHIR, patient recruitment

## Abstract

**Objective:**

Unlocking the data contained within both structured and unstructured components of electronic health records (EHRs) has the potential to provide a step change in data available for secondary research use, generation of actionable medical insights, hospital management, and trial recruitment. To achieve this, we implemented SemEHR, an open source semantic search and analytics tool for EHRs.

**Methods:**

SemEHR implements a generic information extraction (IE) and retrieval infrastructure by identifying contextualized mentions of a wide range of biomedical concepts within EHRs. Natural language processing annotations are further assembled at the patient level and extended with EHR-specific knowledge to generate a timeline for each patient. The semantic data are serviced via ontology-based search and analytics interfaces.

**Results:**

SemEHR has been deployed at a number of UK hospitals, including the Clinical Record Interactive Search, an anonymized replica of the EHR of the UK South London and Maudsley National Health Service Foundation Trust, one of Europe’s largest providers of mental health services. In 2 Clinical Record Interactive Search–based studies, SemEHR achieved 93% (hepatitis C) and 99% (HIV) F-measure results in identifying true positive patients. At King’s College Hospital in London, as part of the CogStack program (github.com/cogstack), SemEHR is being used to recruit patients into the UK Department of Health 100 000 Genomes Project (genomicsengland.co.uk). The validation study suggests that the tool can validate previously recruited cases and is very fast at searching phenotypes; time for recruitment criteria checking was reduced from days to minutes. Validated on open intensive care EHR data, Medical Information Mart for Intensive Care III, the vital signs extracted by SemEHR can achieve around 97% accuracy.

**Conclusion:**

Results from the multiple case studies demonstrate SemEHR’s efficiency: weeks or months of work can be done within hours or minutes in some cases. SemEHR provides a more comprehensive view of patients, bringing in more and unexpected insight compared to study-oriented bespoke IE systems. SemEHR is open source, available at https://github.com/CogStack/SemEHR.

## BACKGROUND

The opportunity for secondary use of the wealth of information contained within electronic health records (EHRs) has attracted researchers interested in investigating approaches to provide more tailored and timely care, improve efficiency of services, and derive new scientific and medical insights.^1–4^ In addition to structured data contained within relational database tables (such as International Classification of Diseases, Tenth Revision [ICD-10] diagnoses codes), EHR documents are filled with unstructured clinical notes, such as nursing records, radiology reports, and discharge summaries. These notes add a richness and depth to EHR-based studies,^5–7^ providing data and insight beyond what is available within the thin layer of data stored within structured fields.

Deriving actionable insights from the EHR, including the unstructured component, is challenging. It requires bringing together expertise in the clinical domain, the underlying health care information systems, and text analytics techniques, eg, natural language processing (NLP). For example, the Clinical Record Interactive Search (CRIS) system,^8^ an anonymized replica of the EHR used in the South London and Maudsley (SLaM) National Health Service (NHS) Foundation Trust in the UK, was designed to support clinical and scientific studies. Since its launch in 2009, a large number of studies (^9–13^ to name a few) have used the CRIS resource in conjunction with NLP or text-mining techniques. Although these studies answered different clinical questions, the technical requirements for extracting, structuring, and making sense of the data largely overlapped, and included (1) preprocessing and cleansing corpus-related documents (eg, removing misleading form headings from scanned documents); (2) compiling and recognizing common medical terminology (eg, the antipsychotic medication identification problem is almost the same in^10^^,^^11^); and (3) deriving patient-level clinical signals from document-level NLP annotations (eg, understanding that a medication prescribed at admission was removed from the patient’s discharge medication list).

As unstructured EHR data are inevitably needed by many research projects and clinical studies, more cost-effective and systematic solutions are needed to address the common challenges presented by different use cases, while also ensuring that study-specific requirements are not compromised by the unified approach.

To address such challenges, we propose SemEHR, a semantic search and analytical system that generates a complete and processable view of patients from their clinical notes.

- To realize a *general-purpose* biomedical information extraction (IE) system for EHRs, there are at least 3 fundamental challenges: (1) syntactic heterogeneity: how to effectively access multimodal/multisource EHR data that are almost certainly heterogeneous in format, data model, and access interface; (2) knowledge coverage: how to cover all possible biomedical concepts that are required by potential use cases; and (3) context capturing: how to represent and capture the contexts associated with extracted concepts and determine which are critical to understand the clinical domain. To address these challenges, SemEHR uses a production infrastructure that integrates our previous work in the CogStack pipeline^14^ to harmonize and cleanse heterogeneous records, using them to identify contextualized mentions (negation, temporality, and experiencer) of a wide range of biomedical concepts, including Systematized Nomenclature of Medicine Clinical Terms (SNOMED CT) (http://www.snomed.org/snomed-ct), ICD-10 (http://apps.who.int/classifications/icd10/browse/2010/en), Logical Observation Identifiers Names and Codes (LOINC) (https://loinc.org/), and Drug Ontology (https://ontology.atlassian.net/wiki/spaces/DRON/overview). In addition, SemEHR automatically associates semantic types of annotations and their clinical contexts (derived from documents or sections) with dedicated extraction rules, which enables better IE capabilities, such as populating structured vital sign data from observation notes.
- It is well appreciated that a one-size-fits-all approach needs to be adapted to work effectively in different scenarios. Therefore, to serve different use cases well, we require the capability to extend the terminology of the general-purpose IE system to cover unseen concepts, deal with language specificities in a subcorpus, support use case–specific extraction requirements, and enable performance fine-tuning, eg, by incorporating specific knowledge or researchers’ expertise. SemEHR provides a study-based (use case–specific) learning engine that enables iterative learning and feedback. It collects user feedback and uses rule-based and machine learning techniques to tackle study-specific challenges and requirements in a continuous manner.
- A few hurdles prevent the effective consumption of extracted data from general-purpose IE systems in scientific research and clinical studies. To fulfill requirements by various studies, developing general-purpose IE systems is inevitable in order to adopt large terminologies that users might not be familiar with. This poses challenges in (1) mapping look-up concepts to terminology terms, (2) translating clinical relations to term associations, and (3) exploiting terminology semantics to bring unexpected or unperceived new insights. At the consumption level, SemEHR implements an ontology-based semantic search component to tackle such challenges.
- Last, and probably most important, EHRs represent a timeline of multiple patient interactions with services. As such, the document-level IE results should be integrated at the patient level to incorporate temporal and macrocontextual information (which reports, which visits, etc., as opposed to the sentence-based contextual information discussed above). Only after this integration is the EHR IE task complete. However, this requires a thorough understanding of the EHR system. SemEHR provides a multiperspective view of each patient by assembling NLP annotations at the patient level as longitudinal views and compiling structured medical profiles. Both the NLP results and the patient timeline are made available via an ontology-based search system, which effectively turns common IE tasks into semantic search queries. The interface provides a multiperspective view of each patient by assembling NLP annotations at the patient level as longitudinal views and compiling structured medical profiles.

## METHOD

### Data model and longitudinal patient views

**Figure 1.**
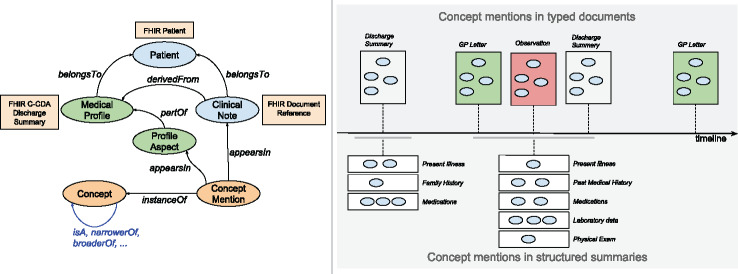
(A) SemEHR data model: entities (patient, clinical note, concept, and concept mentions) and their associations. (B) SemEHR generates 2 longitudinal views for each patient: concept mentions grouped in typed and dated documents (upper part), and concept mentions grouped in structured (discharge) summaries (lower part).

As depicted in Figure 1A, SemEHR is built upon 6 types of entities: patient, clinical note, concept, concept mention, medical profile, and profile aspect. Each *patient* is associated with a list of dated and typed *clinical notes*. From these notes, SemEHR identifies *mentions* of a wide range of biomedical *concepts* from the Unified Medical Language System (UMLS),^15^^,^^16^ a compendium of many controlled vocabularies, including SNOMED CT, ICD-10, LOINC, Drug Ontology, and Gene Ontology. By analyzing the context of its appearance, each *mention* is associated with 3 pieces of dimensional contextual information: negation, temporality, and experiencer. Highlighted in green in Figure 1A, the associations between concepts (eg, *Steatohepatitis is a liver disease; Ribavirin is a drug for treating hepatitis C*) are made available to conduct semantically enriched computations by incorporating the various biomedical ontologies and Linked Open Data (https://en.wikipedia.org/wiki/Linked_data) such as DBpedia^17^ and Wikidata.^18^ SemEHR derives periodical *medical profiles* from a patient’s clinical notes, automatically generated medical summaries consisting of a set of *profile aspects* (sections describing different aspects of a medical profile, eg, past medical history, medications, etc.) for a defined period of time*. Concept mentions* are assigned to these *aspects* according to their appearance in the original clinical notes. As the rectangle boxes in Figure 1A show, SemEHR entities are mapped to Fast Healthcare Interoperability Resources (FHIR) (https://www.hl7.org/fhir/overview.html) entities whenever possible.

Based on this data model, SemEHR populates 2 longitudinal views (shown in Figure 1B) for each patient. As shown in the upper part of Figure 1B, the first view is generated directly from the raw data. Concept mentions are organized in a list of clinical notes that are located on a timeline according to their date attributes (eg, the created date/time of the clinical notes). Wherever possible, types of clinical notes (such as GP Letter, Radiology, or Discharge Summary) are presented.

The second view (lower part of Figure 1B) is designed to convey structured summaries for a patient, each of which summarizes the patient’s medical history/conditions in a period of time (eg, an inpatient hospital stay). A summary is composed of groups of concept mentions, where each group is about one particular aspect of the patient’s medical profile, eg, past medical history, medications, or physical exams. Preferably, such summaries are derived from discharge summaries. When discharge summaries are not available, an automated summarization approach is applied to generate the summaries based on the contextual information of the concept types and concept mentions. Automated summaries are differentiated from those generated from discharge summaries. Supplementary Material 2 describes the detailed process of automated medical profile generation.

### Architecture: generic and adaptive information extraction and retrieval

**Figure 2.**
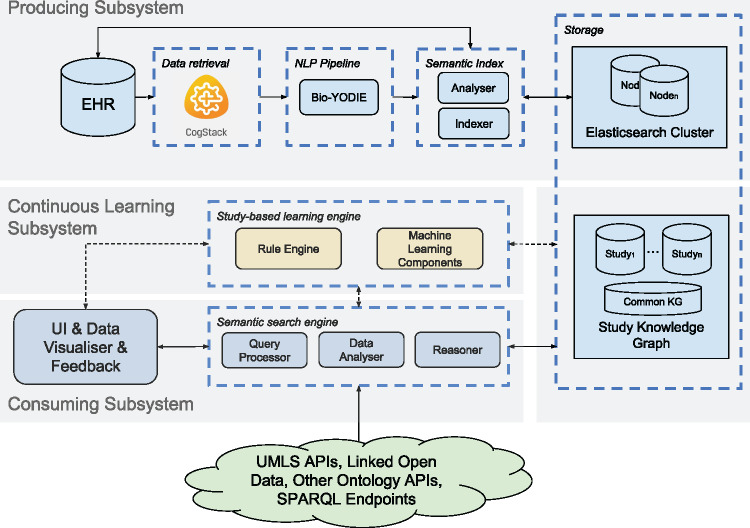
The architecture of SemEHR is composed of 3 subsystems: (1) the producing subsystem (upper part of the figure), creation of SemEHR semantic index by harmonizing, natural language processing, and indexing EHR data; (2) the continuous learning subsystem, addressing study-specific requirements and supporting fine-tuning for separate studies; and (3) the consuming subsystem (lower part), supporting tailored care, patient recruitment, and clinical research by semantic searching and study-based continuous learning.

As illustrated in Figure 2, SemEHR is composed of 3 subsystems: the producing subsystem, the continuous learning subsystem, and the consuming subsystem.

#### The producing subsystem

Essentially, the producing subsystem extracts free-text clinical notes from heterogeneous underlying EHR systems, populating the data model described in the previous section. This task is performed in 3 main steps: data retrieval, information extraction, and semantic indexing. CogStack,^14^ a data harmonization and enterprise search toolkit for EHRs, is adopted in the data retrieval step to provide a unified interface with unstructured EHR data, which is often very heterogeneous in format and distributed in storage. Each document that flows out from the data retrieval component is fed into the NLP pipeline, which embeds Bio-YODIE (https://gate.ac.uk/applications/bio-yodie.html), an NLP pipeline dedicated to annotating UMLS concepts in clinical notes (“documents” hereafter). (Bio-YODIE was developed as part of the EU KConnect project, in which GG, AR, HW, RS, and RD are involved.) Emerging from the NLP pipeline are the documents and concept mentions extracted from them, which are then analyzed by the Semantic Index component before being indexed. The analysis involves deriving document types (eg, Radiology, GP Letter, or Discharge Summary), parsing document structure (eg, identifying headed blocks from discharge summaries), and associating concept mentions with document structures. The analysis results, document content, and NLP outputs are finally indexed by an Elasticsearch (https://www.elastic.co/products/elasticsearch) cluster. Patient-level summaries are generated as described in the previous section. These summaries are updated as new documents are added to the index.

SemEHR aims to produce annotations with accurate contextual information. Three components work collectively to achieve this goal: the Bio-YODIE pipeline captures the sentence/paragraph-level contexts (eg, negation, hypothetical mentions); the semantic index’s analyzer brings in section/document-level context (eg, past medical history, laboratory results); and the continuous learning subsystem (described in the next subsection) learns the contexts from user-assessed annotations (see Supplementary Material 1 for details).

#### The continuous learning subsystem

To accommodate the uniqueness of the IE requirements of different studies, SemEHR is designed with a continuous learning subsystem to iteratively address study-specific issues. The system collects and analyzes user feedback from an annotation component embedded within the user interface. Based on the analyzed feedback, 2 components are used to improve the IE results. The first is a rule engine, which generates and applies rules for filtering out unwanted results, eg, removing concept mentions based on their original string or surrounding text. The second component is a machine learning engine (a bidirectional recurrent neural network model), which takes user feedback as training data, applies the trained model on the study’s corpus, and populates a confidence value for each concept mention. Confidence values are used as quantified indicators in analytic components for populating results. The user interface for collecting feedback and the continuous learning mechanisms are explained in detail in Supplementary Material 1.

#### The consuming subsystem

This subsystem consists of a set of components that utilize IE results and clinical knowledge (accessed from biomedical ontology and Linked Open Data application programming interfaces) to support tasks such as patient characterization or trial recruitment. A consuming task is called a “study” in SemEHR. Each study will have its own storage within SemEHR’s Study Knowledge Graph (KG) (bottom of the Storage section in Figure 2), which stores its study parameters (eg, cohort definition and metadata), search settings (eg, query concepts), study results (eg, selected cohort and exported features), and customized rules (eg, regular expressions to remove unwanted annotations). There is also a common KG (Common KG in Figure 2), where sharable knowledge or efforts (such as manually selected concepts of alcohol-related liver diseases or postprocessing rules for improving NLP results) are made available to other studies.

**Figure 3.**
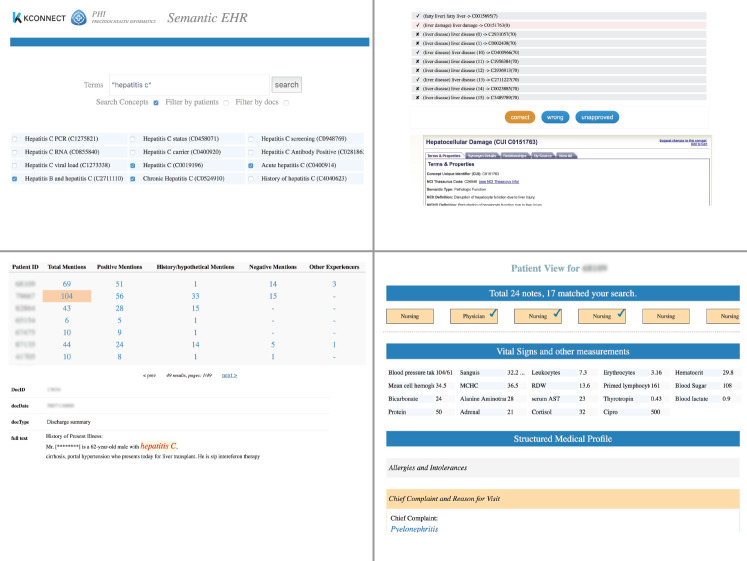
Screenshots of key functionalities provided by the consuming subsystem. (A) Identifying query concepts (UMLS CUIs): facilities to ensure the correct and complete concepts are used in the query to derive accurate clinical findings. (A1) Concept search for matching a user search term to one or more ontology (UMLS) concepts; logical reasoning is implemented to enable the automated inclusion of semantically related concepts (eg, hepatocellular damage is liver damage). (A2) Concept validation component for checking and approving the automated inferred concepts based on the aim and criteria of the clinical study (eg, only retain alcohol-related liver conditions for addiction analytics). (B) Selecting and summarizing cohort (the full text in the screenshot has been deliberately rewritten to avoid leaking sensitive patient data). A summary table is generated for a user query where each row summarizes the numbers of total mentions and contextualized mentions for one patient. (C) Patient timeline: longitudinal document view (upper), structured medical profile view (based on FHIR discharge summary format), and the view of latest vital signs and other measurements.

Key functionalities of the consuming subsystem include the following:

- *Translating search terms to query concepts.* This translates the user’s keyword searches (which are often ambiguous or incomplete) into semantically clear concepts (identified using UMLS Controlled Unclassified Information). The correct translation is essential to ensure the soundness and completeness of search and analytics results. Unfortunately, in the clinical scenario, it is often not a trivial task to compile an accurate and complete concept list even for a single clinical signal. For example, one SemEHR case study needs to look up patients with alcohol-related liver disease. Given a general clinical term such as “liver disease,” it would be time-consuming to compile a list of all subtypes of liver disease that are also alcohol-related. As depicted in section A of Figure 3, SemEHR provides 2 functions for supporting concept translation: (1) matching search terms to concepts, which is enhanced with logical reasoning to automatically include semantically related concepts and EHR-based exclusion to remove concepts that do not exist in EHRs of the study cohort; and (2) validating automatically populated lists, to allow manual assessment by the researchers.
- *Selecting and summarizing a cohort.* Each query submitted to SemEHR will result in a cohort, a list of patients who match the query. As shown in Figure 3B, a summary table is generated for the matching cohort. Each row summarizes a patient, and the first column shows the patient ID. The second one shows the total number of mentions of the search concepts within this patient’s EHR, followed by numbers of 4 contextualized mentions: positive mentions, history/hypothetical mentions, negated mentions, and mentions associated with other experiencers. Clicking on the numbers brings the user to the clinical notes, where corresponding mentions are highlighted (lower part of Figure 3B).
- *Generating patient views and structured medical profile.* As a generic IE and retrieval platform, SemEHR processes all EHR records for patients and tries to identify a wide range of biomedical concepts from them. This enables it to produce a panorama for each patient. As shown in Figure 3C, 3 different views are generated for each patient:
  - The first view is the longitudinal document view (upper part of Figure 3C), which lists all patient documents in chronological order, labels documents using their types, and ticks those documents that match the query. This view delivers the abundance of a patient’s records, the prevalence of matched documents, and their temporal distributions.
  - The second view is the structured medical profile (lower part of Figure 3C), which is automatically derived from the patient’s clinical notes and structured using extended FHIR discharge summary format (23 sections of the FHIR discharge summary [http://hl7.org/fhir/us/ccda/2017Jan/StructureDefinition-CCDA-on-FHIR-Discharge-Summary.html] are extended with an additional 8 headings). This structured summary enhances SemEHR’s search and IE ability. For example, by constraining the search field to “Family History,” one can get a cohort of patients with a family history of a certain disorder. In addition, knowing that a piece of text appeared in the “Hospital Discharge Physical,” sophisticated rules can be applied to extract more structured data, such as vital signs.
  - The third view is the view of vital signs and other measurements (middle part of Figure 3C). This is automatically generated by applying IE rules on the latest structured summary of a patient.

Based on these key functionalities, SemEHR provides a set of search interfaces to surface the clinical variables hidden in clinical notes. A typical query, such as “return all patients with a family history of hepatitis C,” previously might have required the end user to have NLP expertise, eg, be able to do named entity recognition for “hepatitis C” that must be mentioned in the context of “family history.” Using SemEHR, the end user can put in a simple keyword search: “hepatitis C.” To fulfill this search, SemEHR will pull out the cohort of relevant patients, populate patient-level summaries (ie, numbers of contextualized concept mentions, such as patient has 16 total mentions of the disease, 15 of them positive and 1 about a family member), and provide a link to each mention in the original source clinical note (similar to the UI illustrated in Figure 3B).

## RESULTS

This section reports the experiments and results from 3 EHR systems focusing on evaluating SemEHR’s capacities in semantic search, analytics, and clinical decision-making support. The evaluation on its natural language processing (Bio-YODIE) performance is available in Supplementary Material 3.

### Studies conducted on CRIS data of South London and Maudsley Hospital

SemEHR has been deployed on the anonymized psychiatric records database CRIS,^8^ which contains a total of 18 million free-text documents from South London and Maudsley Hospital, one of Europe’s largest mental health providers (serving 1.2 million residents). In the CRIS clinical notes, SemEHR identified 46 million mentions of concepts, the predominant ones being pharmacologic substances (16 million), mental or behavioral dysfunction (12 million), and sign or symptom (3.8 million). In a CRIS-based liver disease study, SemEHR identified (in the context of an information retrieval task) 94 instances out of 100 hepatitis C–positive patients that were manually annotated (based on structured blood test data). In an HIV study, a random 1000-patient cohort was selected, and SemEHR identified 21 out of 23 true positive (verifiable via structured blood test data) HIV patients using 2 search concepts, HIV Pos (UMLS code: C0019699) (20 true positives) and HIV diagnosis (UMLS code: C0920550) (8 true positives). SemEHR integrates document-level NLP annotations at the patient level to generate an integral view of patients. Table 1 presents the results of 2 experiments designed to evaluate the effectiveness of such integration on 2 case studies, hepatitis C and HIV. The results show that the number of positive mentions of diseases at the patient level is a good feature for supervised learning methods (naive Bayes or decision table) to classify whether a patient suffers from a disease or not. (The results reported in Table 1 are of a *classification* task, which is different from the previous information retrieval task.)

**Table 1.** Given a disease (identified by one or more UMLS concepts, ie, search concepts), SemEHR can generate a summary table for a cohort of patients, which, for each patient, gives the number of positive mentions of the search concepts within all of his/her EHR documents. Using this number as the only feature, we classify whether a patient suffers from a disease or not.

	Precision	Recall	*F*-measure	Class (200)^a^		Precision	Recall	*F*-measure	Class (1000)^b^
	0.857	0.522	0.649	Hepatitis C positive (33)		0.985	0.855	0.915	HIV positive (76)
	0.941	0.989	0.964	Hepatitis C unknown (177)		0.988	0.999	0.994	HIV unknown (924)
Weighted avg.	0.931	0.935	0.928		Weighted avg.	0.988	0.988	0.988	

^a^Two hundred CRIS patients evaluated for hepatitis C; classification model: *naive Bayes*; test method: 10-fold cross-validation; search concepts: C0019196, C2148557, C0220847. This shows the results of a 200-patient cohort for hepatitis C infection.

^b^One thousand CRIS patients evaluated for HIV; classification model: *decision table*; test method: 10-fold cross-validation; search concepts: C0019699, C0920550. This shows the results of a 1000-patient cohort for HIV.

### Study conducted at King’s College Hospital, London

At King’s College Hospital, SemEHR is being used to assess eligibility and subsequently recruit patients into the 100 000 Genomes Project (https://www.genomicsengland.co.uk/). Here, an open SPARQL endpoint is integrated to map UMLS concepts to Human Phenotype Ontology terms, inclusion criteria for recruitment, and the concepts necessary to populate complex phenotype models. The preliminary validation study suggests that the tool is able to validate previously submitted cases and is very fast at searching phenotypes (providing results within seconds), an operation that previously required manual assessment of patient records. For example, the time to check the recruitment criteria for a patient is reduced significantly from days to minutes for dermatology disorders, for which the inclusion/exclusion criteria contain 120 phenotypes, on average. In addition, semantic reasoning (eg, expanding search concepts with more specific concepts) has been found to be helpful for identifying 2 specific phenotypes, neutropenia and hypertension.

### Studies conducted on MIMIC-III data

We deployed a SemEHR instance on MIMIC-III,^19^ an intensive care EHR dataset anonymized from 2 US-based hospitals and made public for research purposes. MIMIC-III contains about 2 million free-text clinical notes and comprises very good structured data, including high-resolution laboratory measurements for most patients. To evaluate the performance of SemEHR’s structured medical profile, we randomly selected 100 patients and assessed the accuracy of automatically extracted laboratory measurements in their SemEHR medical profiles. The results are presented in Table 2. Eleven types of laboratory measurements were manually selected for this evaluation, which contains popular tests such as hematocrit and relatively rare ones such as blood urea. First, we compared the extracted measurement values with those stored in the MIMIC-III structured data. A patient usually has multiple values of the same measurement that are tested at different times, and it should be noted that as long as the extracted value appears within the list of all values from the structured data, the extraction is deemed correct; otherwise, it is incorrect. The result of the first step is presented in the second to last column. The average accuracy using structured data verification is 89%. For those incorrect extractions, we applied a second step of manual assessment. This step identified some false negative results from the first step caused by factors such as decimal rounding (3 cases), different units (2 cases), and missing laboratory events in structured data (6 cases). The accuracies based on the manual verification are reported in the last column of Table 2. The average accuracy was improved to 97%.

**Table 2.** The performance of SemEHR laboratory measurement extraction on MIMIC-III data: 11 measurements are studied (first column); 100 patients were randomly selected for this study

Laboratory measurements (UMLS label)	MIMIC-III label	# Correct (structured data comparison)	# Incorrect (structured data comparison)	# Actually correct (manually verified)	# Total extracted measurements	Accuracy (structured data comparison) (%)	Accuracy (manually verified) (%)
Hematocrit	Hematocrit	38	5	4	43	88.37	97.67
Platelets	Platelet count	1	1	1	2	50.00	100.00
Sodium	Sodium	15	0	0	15	100.00	100.00
Mean corpuscular hemoglobin concentration	Mean corpuscular hemoglobin concentration	35	1	0	36	97.22	97.22
Alanine aminotransferase	Alanine aminotransferase	19	3	2	22	86.36	95.45
Red blood cell distribution width	Red blood cell distribution width	35	1	0	36	97.22	97.22
Serum aspartate aminotransferase	Aspartate aminotransferase	20	2	1	22	90.91	95.45
Chloride	Chloride	15	0	0	15	100.00	100.00
Blood urea	Urea nitrogen	3	0	0	3	100.00	100.00
Leukocytes	White blood cells	34	5	4	39	87.18	97.44
Glucose	Glucose	18	3	0	21	85.71	85.71
Average accuracy						89.36	96.93

The extracted results were assessed by 2 steps: (1) comparing with the structured data (querying lab events table in MIMIC-III; accuracy reported in the 7th column), and (2) manually checking not-matched items in the first step (accuracy reported in the last column).

The manual verification revealed that extracting vital signs from clinical notes can complement structured data in MIMIC-III; there are 6 cases where the measurements are extracted from free text but missing in the structured data. In general, SemEHR can reveal various types of structured data that usually are not or cannot be recorded in structured EHRs, such as family history and social history. In Table 3, for the above 100 randomly selected patients, we summarize the number of semantic entities identified in 5 sections of SemEHR medical profiles which are usually not recorded in structured EHRs.

**Table 3.** The number of extracted semantic entities in 5 sections of SemEHR medical profiles of the 100 randomly selected MIMIC-III patients, which are usually not recorded in structured EHRs

Admission medications		Family history		Social history		History of past illness		Hospital discharge instructions	
# Total annotations									
1475		156		445		1575		1162	
Top 5 semantic types by frequency									
Temporal concept	442	Finding	42	Finding	132	Disease or syndrome	337	Clinical attribute	359
Pharmacologic substance	393	Disease or syndrome	33	Temporal concept	86	Finding	189	Temporal concept	158
Finding	194	Neoplastic process	28	Pharmacologic substance	58	Temporal concept	182	Health care–related organization	133
Clinical drug	121	Pharmacologic substance	14	Clinical attribute	30	Therapeutic or preventive procedure	180	Health care activity	126
Health care–related organization	51	Clinical attribute	9	Individual behavior	28	Body part, organ, or organ component	96	Finding	79

## DISCUSSION

SemEHR has been deployed or is in the process of being deployed in a number of NHS Trust EHR systems, including South London and Maudsley, King’s College Hospital, University College London Hospitals, and Guy’s Hospital. Results and feedback from the multiple SemEHR use cases have shown its effectiveness in automating lengthy manual tasks without jeopardizing accuracy. Queries are returned at a rapid enough rate to enable iterative tailoring to achieve high specificity. Moreover, according to our case studies at SLaM, SemEHR has achieved similar accuracy to bespoke NLP applications built upon TextHunter.^13^ With a system powered by ontological semantics, researchers can make use of semantically associated concepts to improve results, eg, in the CRIS-based liver disease study, the inclusion of 8 drugs used for treating liver disease helped to find more patients.

Our case studies show that building a unified framework like SemEHR realizes a more cost-effective approach to dealing with common IE challenges and significantly lowers the barrier for researchers, coders, and clinicians to access knowledge residing within unstructured clinical notes. SemEHR has great potential in enabling the efficient and effective secondary use of EHRs to improve health care services. Furthermore, SemEHR-like systems initiate a collaborative learning platform, as advocated by Moseley and et al.,^20^ enabling studies to be conducted in a cooperative way rather than having resources remain in isolated silos.

SemEHR provides different patient views, with the aim of presenting a more continuous representation of the patient’s treatment timeline. Such views may reveal data quality issues^21^^,^^22^ to researchers or clinicians so that necessary actions can be taken before deriving conclusions. For example, the longitudinal document view gives a quick overview of how abundant or detailed a patient’s EHR is, which helps to identify patients who have incomplete records and might need to be removed from studies. However, data quality issues such as data incompleteness, inconsistency, and inaccuracy need to be addressed in a systematic way; making users aware of the potential issues is only the first step. In our future work, we will investigate approaches to tackling challenges such as checking automated patient-level consistency, bearing in mind that some of the challenges require wider-scope (eg, institution-level) attention.^23^^,^^24^

## CONCLUSION

In this paper, we presented SemEHR, a unified information extraction and semantic search system for obtaining clinical insight from unstructured clinical notes. With a dedicated architecture and the incorporation of semantic analytics, SemEHR effectively turns IE tasks into (iterative) ontology-based searches, which significantly lowers the barriers to secondary use of unstructured EHR data. The system has been deployed in several NHS hospitals in the UK and a number of case studies have been initiated, including patient recruitment for the UK government’s 100 000 Genomes Project. Results and feedback demonstrate that SemEHR can efficiently perform the task of cohort selection and patient characterization with high accuracy. SemEHR is open source; all nonsensitive data relating to its verifications have been published in its online repository: https://github.com/CogStack/SemEHR.

## FUNDING STATEMENT

This work was supported by the Medical Research Council (grant number MC_PC_14089 and MR/L014815/1), the National Institute for Health Research Biomedical Research Centre at South London and Maudsley NHS Foundation Trust and King's College London, the European Union’s Horizon 2020 (grant number 644753 KConnect), the Wellcome Trust Seed Award in Science (grant number 109823/Z/15/Z), the National Institute for Health Research University College London Hospital’s Biomedical Research Centre, Arthritis Research UK, the British Heart Foundation, Cancer Research UK, the Chief Scientist Office, the Economic and Social Research Council, the Engineering and Physical Sciences Research Council, the National Institute for Social Care and Health Research, and the Wellcome Trust (grant number MR/K006584/1).

## COMPETING INTERESTS STATEMENT

We declare no competing interests.

## CONTRIBUTORSHIP STATEMENT

HW, IK, AF, AR, GG, RJ, ZI, and AA were involved in development of SemEHR or components that are used by the system. GT and KIM led the liver disease and HIV studies on the SLaM EHR. RS led an autoimmune study on the SLaM EHR; CS and DG led the 100 000 Genomes Project study at King’s College Hospital. MB and RS provided the access and computational resources for accessing the SLaM EHR. AR, RS, and RJBD secured funding for this research. All authors contributed to the abstract.

## Supplementary Material

Supplementary Data
