# Supplementary material for: SemEHR: A general-purpose semantic search system to surface semantic data from clinical notes for tailored care, trial recruitment, and clinical research
Source: J Am Med Inform Assoc. 2018 Jan 19;25(5):530–7. doi: 10.1093/jamia/ocx160 (PMC6019046; doi:10.1093/jamia/ocx160)
Supplement: Supplementary Data [file ocx160_supp.docx]

## Supplementary material 1: user feedback and continuous learning

| 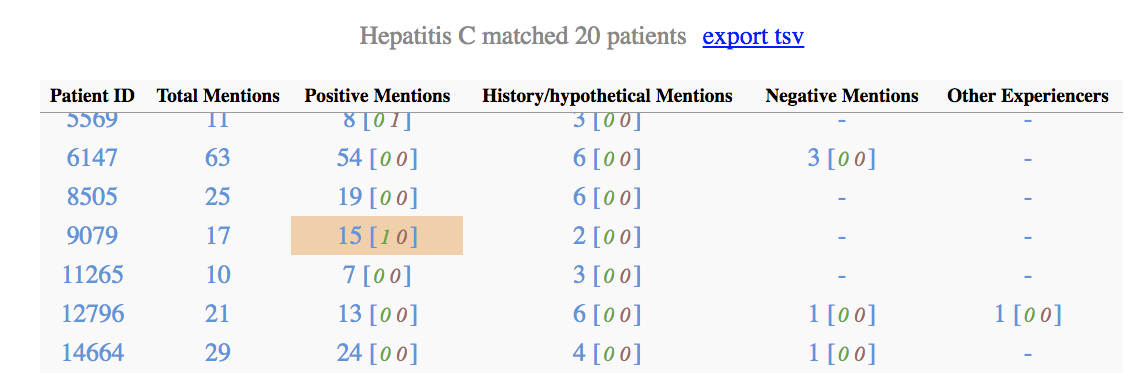  *Figure S1a. The summary table of current assessed results: each row presents the number of annotations for a patient; the last four columns are the numbers of typed mentions; there is a bracket beside each typed annotation number, which contains two numbers that indicate the assessment results - the first number (in green) is the number of correct annotations and the second one (in brown) is that of the incorrect ones. Clicking on numbers will instruct the interface to bring up corresponding annotations that are highlighted within their containing clinical note(s) as shown in Figure S1b.* |
| --- |
| 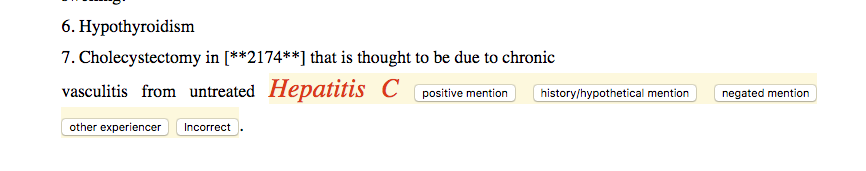  *Figure S1b. The clinical notes with the annotation(s) in question highlighted. Each annotation is provided with 5 pre-given labels: positive mention, history/hypothetical mention, negated mention, other experiencer and incorrect. The user can choose one of the labels according to his/her own understanding. The result will be saved to the server on the fly and summary table (Figure S1a) will be updated accordingly.* |

Figure S1. Feedback by manual annotation assessment: SemEHR provides a feedback mode, where the system randomly select a given number of patients that match the search and users are allowed to assess each annotation by selecting the correct label from the five pre-given ones.

As shown in Figure S1, SemEHR provides a user feedback mode, where the users can assess the annotations by selecting correct labels for them manually. The set of annotations to be assessed is randomly selected.

The assessment results can be utilised in two ways. In the first approach, the labelled data will be used by a recurrent neural network model, which can learns a confidence value for annotations in this particular search task. A preliminary result has shown that such model can help improve the accuracy near 25% in the liver disease use cas at SLaM. In the second approach, incorrect annotations will be studied by the researchers and clinicians together and rules will be manually crafted for a post process step to further improve the performance for this particular search task.

## Supplementary Material 2: automated structured medical profile generation


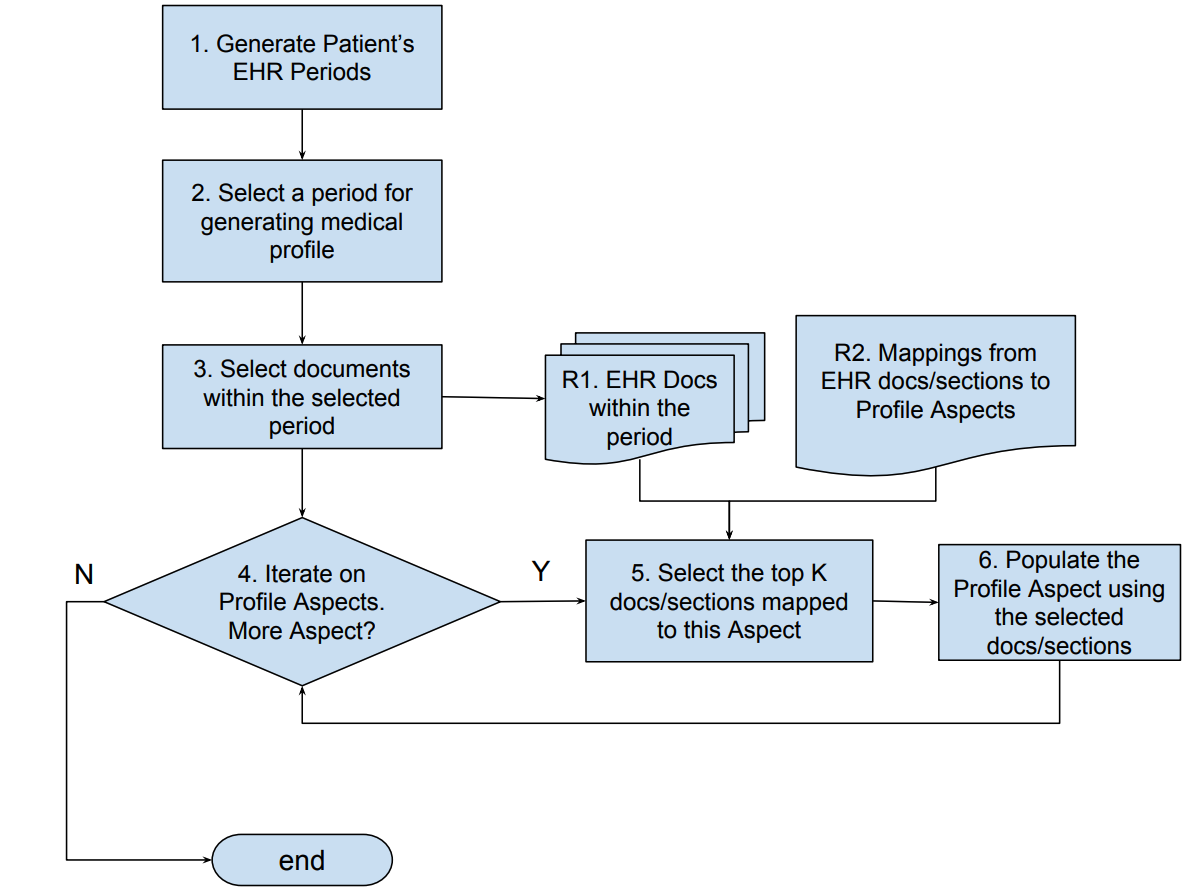


Figure S2. The process of automated structured medical profile generation when discharge summaries are not available

When discharge summaries are not available, SemEHR will automatically populate structured medical profile for a patient using the process as described in Figure S2. The descriptions for steps are given as follows.

1. Patient’s EHR timeline is separated into periods based on the time gaps between documents: if the gap is larger than a given threshold (a 30 day threshold is used by SemEHR now), a new period will be generated.
2. Pick up a period from the list periods to generate structured medical profile for.
3. Select a list of EHR documents within a selected period of time for processing. A resource (identified as R1 in Figure S2) is generated for later use.
4. To populate the profile, SemEHR iterates on the list of Profile Aspects (e.g., Discharge Medications, Social History and etc.) as defined in SemEHR’s data model.
5. Two resources are used in this step for populating a Profile Aspect. R1 is the list of documents from step 3. R2 is the mappings defined for each EHR system that associate EHR document types or document sections to Profile Aspects. With R1 and R2, for each Profile Aspect, the system picks up the documents or sections of documents that are mapped to this Aspect. If there are more than one documents or sections of documents, it will choose the top K based on a ranking algorithm (at this moment, K is 1 and the ranking algorithm is just picking up the most recent document or document section).
6. The selected documents or document sections are used to populate the Profile Aspect. If there is no document available, this Aspect will be left as blank.

## Supplementary Material 3: Bio-YODIE evaluation on ShARe Corpus

| **System** | **Secs** | **Precision** | **Recall** | **F1** | **Accuracy** | **Scott's Pi** |
| --- | --- | --- | --- | --- | --- | --- |
| MetaMap | 3811 | 0.574 | 0.568 | 0.571 | 0.857 | 0.856 |
| MetaMapLite | 986 | **0.654** | 0.549 | **0.597** | 0.877 | 0.876 |
| Bio-YODIE | **573** | 0.582 | **0.605** | 0.593 | **0.883** | **0.882** |

Table S1. Evaluation of Bio-YODIE on ShARe Corpus with comparisons to MetaMap and MetaMapLite

The experiment was conducted using the ShARe Corpus^^[[1]](#footnote-1)^^, which was built upon the MIMIC II database, version 2.5 (mimic.physionet.org). We compared Bio-YODIE (the NLP component used in SemEHR), MetaMap^^[[2]](#footnote-2)^^ and MetaMap Lite^^[[3]](#footnote-3)^^. The observation is that:

- Bio-YODIE compares favourably to MetaMapLite;
- Unlike MetaMapLite, which relies on fixed rules and models, Bio-YODIE uses corpus priors. This means it can be improved further with more data.

1. https://healthnlp.hms.harvard.edu/share/wiki/index.php/Main_Page [↑](#footnote-ref-1)
2. Aronson, Alan R. "Effective mapping of biomedical text to the UMLS Metathesaurus: the MetaMap program." Proceedings of the AMIA Symposium. American Medical Informatics Association, 2001. [↑](#footnote-ref-2)
3. Demner-Fushman, Dina, Willie J. Rogers, and Alan R. Aronson. "MetaMap Lite: an evaluation of a new Java implementation of MetaMap." Journal of the American Medical Informatics Association (2017): ocw177. [↑](#footnote-ref-3)
